# High risk of osteoporosis and fracture following solid organ transplantation: a population-based study

**DOI:** 10.3389/fendo.2023.1167574

**Published:** 2023-05-23

**Authors:** Hsin Chen, Yung-Rung Lai, Yih Yang, Shuo-Yan Gau, Cheng-Yang Huang, Tung-Han Tsai, Kuang-Hua Huang, Chien-Ying Lee

**Affiliations:** ^1^ School of Medicine, Chung Shan Medical University, Taichung, Taiwan; ^2^ Department of Medical Education, Linkou Chang-Gung Memorial Hospital, Taoyuan City, Taiwan; ^3^ Department of Pharmacology, Chung Shan Medical University, Taichung, Taiwan; ^4^ Department of Pharmacy, Chung Shan Medical University Hospital, Taichung, Taiwan; ^5^ Department of Obstetrics and Gynecology, E-Da Hospital, I-Shou University, Kaohsiung, Taiwan; ^6^ Department of Health Services Administration, China Medical University, Taichung, Taiwan

**Keywords:** solid organ transplantation, hazard ratios, osteoporosis, fracture, cohort study

## Abstract

**Background:**

Osteoporosis and fractures increase morbidity and mortality rates after solid organ transplantation (SOT), but few studies have analyzed the risk of osteoporosis and related fractures after SOT. In this retrospective cohort study, we investigated the risk of osteoporosis and fractures in different SOT recipients.

**Methods:**

This study was a retrospective cohort study using a nationally representative database in Taiwan. We collected the data of SOT recipients and used the propensity score matching method to obtain a comparison cohort. To reduce bias, we excluded patients who had been diagnosed with osteoporosis or fracture before inclusion. All participants were followed up until the date of diagnosis as having a pathological fracture, death, or the end of 2018, whichever occurred first. The Cox proportional hazards model was used to investigate the risk of osteoporosis and pathological fracture in SOT recipients.

**Results:**

After adjustment for the aforementioned variables, SOT recipients were observed to have a higher risk of osteoporosis (hazard ratio (HR) = 1.46, 95% confidence interval (CI): 1.29–1.65) and fracture (HR: 1.19, 95% CI: 1.01–1.39) than the general individuals. Among the different SOT recipients, the highest risk of fractures was noted in heart or lung transplant recipients, with a HR of 4.62 (95% CI: 2.05–10.44). Among the age groups, patients aged >61 years had the highest HRs for osteoporosis (HR: 11.51; 95% CI, 9.10–14.56) and fracture (HR: 11.75, 95% CI: 8.97–15.40).

**Conclusion:**

SOT recipients had a higher risk of osteoporosis and related fractures than the general population, with the highest risks observed in patients receiving heart or lung transplants, older patients, and patients with CCI scores of >3.

## Introduction

Solid organ transplantation (SOT) has been an established treatment for end-stage organ failure (1), including that of the kidney, liver, heart, and lung, over the past few decades (2). Compared with the healthy population, SOT recipients have a high risk of osteoporosis and fracture (3, 4).

Pretransplant bone mineral density (BMD) is considered an important determinant of subsequent osteoporosis development (5). Fractures are very common in patients referred for lung transplantation. Patients referred for lung transplantation have a high pretransplant prevalence of low BMD and osteoporotic fracture, further increasing their posttransplant fracture risk (6). BMD decline primarily occurs at the lumbar spine and femoral neck during the first year after transplantation (7). Typically, BMD decreases rapidly within the first 6 to 12 months after transplantation, accompanied by a marked increase in bone resorption (2). Posttransplantation bone disease is therefore a common and serious complication (8), with osteoporosis occurring in up to half of transplant recipients and vertebral fractures occurring in almost a third (9). Fractures commonly occur after SOT (7). Osteoporosis and fragility fractures significantly affect the quality of life and survival of SOT recipients (10–12).

Osteoporosis and fractures have been associated with considerable disability and are an important cause of morbidity and mortality after SOT. However, the risk of osteoporosis and fracture following SOT has remained unclear. In particular, large-scale epidemiological studies have examined osteoporosis and fracture risk among SOT recipients, especially based on a nationwide database (13). In this retrospective cohort study, we investigated the risk of osteoporosis and related fractures in different recipients of SOT from the National Health Insurance Research Database (NHIRD) in Taiwan from 2001 to 2018.

## Materials and methods

### Data sources

We analyzed the data from the NHIRD, maintained by the Health and Welfare Data Science Center (HWDC), Ministry of Health and Welfare (MOHW), Taiwan. The NHIRD includes details of beneficiaries enrolled in Taiwan’s National Health Insurance (NHI) program from 2001 to 2018, including NHI enrollment files and medical service data (diagnoses, prescription drugs, and examinations). The NHI program is a compulsory single-payer healthcare system providing comprehensive healthcare for >99% of the residents of Taiwan. The diagnostic data were coded using the *International Classification of Diseases, Ninth Revision, Clinical Modification* (*ICD-9-CM*) before 2016 and *ICD-10-CM* after 2016. The NHIRD can serve as a foundation for the procurement of real-world evidence to support clinical decisions and healthcare policy-making (14–17).

### Ethics approval

This study was conducted in accordance with the Declaration of Helsinki. This study protocol was approved by the Central Regional Research Ethics Committee of China Medical University, Taiwan (No. CSMUH CS2-21134). The data used in the present study are anonymous to protect the privacy of beneficiaries. Informed consent was not required given that the database in this study contains only de-identified data.

### Study participants

**Figure 1:**
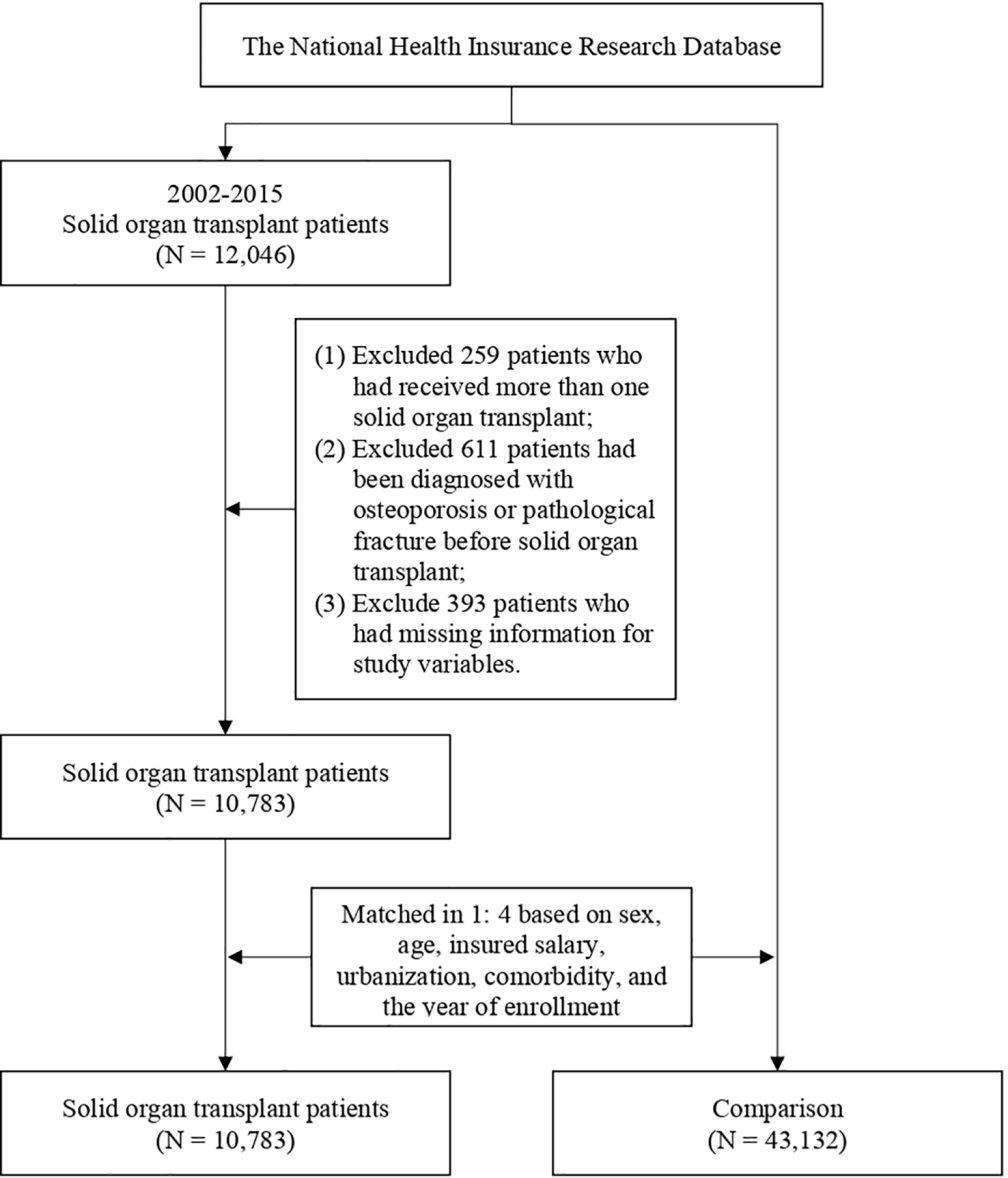
Patient selection process.

We included the data of SOT recipients between 2002 and 2015, including those patients with renal (*ICD-9-CM* V42.0), liver (*ICD-9-CM* V42.7), heart (*ICD-9-CM* V37.51), or lung (*ICD-9-CM*: V42.6) transplantation. We excluded patients who had received more than one SOT, had been diagnosed as having osteoporosis or pathological fracture before SOT, and had missing information for study variables. Osteoporosis was identified using the following codes: *ICD-9-CM* 733.0 and *ICD-10-CM* M810, M816, and M818. The pathological fracture was identified using the following codes: *ICD-9-CM* 733.1 and *ICD-10-CM* M485, M800, M808, and M843–M846. The comparison cohort was made up of general patients who had no diagnosis of osteoporosis or pathological fracture before inclusion. To reduce selection bias, we used 1:4 propensity score (PS) matching for each patient with SOT to obtain a comparison cohort matched for sex, age, insured salary, urbanization, Charlson comorbidity index (CCI), and year of inclusion in the study. After matching, 10,783 SOT recipients and 43,132 controls were included in the study. The patient selection flowchart is presented in **Figure 1**.

### Study design

This study was a retrospective cohort study to examine the risk of osteoporosis and fracture in SOT recipients. The date of SOT was defined as the observation start date for SOT recipients. In terms of investigating the risk of osteoporosis, all participants were followed up until the date of diagnosis as having osteoporosis, death, or the end of 2018, whichever occurred first. In terms of investigating the risk of pathological fracture, all participants were followed up until the date of diagnosis as having a pathological fracture, death, or the end of 2018, whichever occurred first. The comorbidities in the study were defined with the outpatient department visits and hospital admission database in the past 2 years before the observation date. The comorbidities included hypertension (HTN) (*ICD-9-CM* 401-405; *ICD-10-CM* I13 and I15), left ventricular hypertrophy (LVH) (*ICD-9-CM* 429.3; *ICD-10-CM* I51.7), atrial fibrillation (AF) (*ICD-9-CM* 427.31; *ICD-10-CM* I48.0, I48.2, I48.91), hyperlipidemia (HPL) (*ICD-9-CM* 272; *ICD-10-CM* E78.4 and E78.5), rheumatoid arthritis (RA) (*ICD-9-CM* 714; *ICD-10-CM* M05-M06 and M45), depression (*ICD-9-CM* 311; *ICD-10-CM* F32.9), sleep disturbance (*ICD-9-CM* 307.4, 780; *ICD-10-CM* G47.8 and G47.9), gout (*ICD-9-CM* 274.9; *ICD-10-CM* M10.9), chronic obstructive pulmonary disease (COPD) (*ICD-9-CM* 490–492 and 494–496; *ICD-10-CM* J40–J44 and J47), hyperthyroidism (*ICD-9-CM* 242.9; *ICD-10-CM* E05.90), chronic kidney disease (CKD) (*ICD-9-CM* 585; *ICD-10-CM* N18), and diabetes mellitus (DM) (*ICD-9-CM* 250; *ICD-10-CM* E08-E13).

### Statistical analysis

All statistical analyses in the study were conducted using SAS version 9.4. The difference in baseline characteristics between SOT recipients and the comparison cohort was analyzed *via* the Chi-square test and Fisher’s exact test. The Cox proportional hazards model was used to investigate the risk of osteoporosis and pathological fracture in SOT recipients with adjustment of the relevant variables, and the results are presented as hazard ratios (HRs) with 95% CIs. *p* <.05 was set as statistically significant.

## Results

**Table 1** presents the basic characteristics of SOT recipients and a comparison after matching. PS matching yielded 53,915 participants, with 43,132 (80%) men and 10,783 (20%) women. The mean age of the SOT recipients was 47.12 ± 14.44 years and that of the controls was 48.80 ± 15.30 years. After PS matching, no between-group differences were observed in sex, age, insured salary, urbanization, and CCI (all *p* > 0.05). Among SOT recipients, 5,092 had HTN (47.22%), 2,123 had CKD (19.69%), 1,667 had HPL (15.46%), 1,259 had sleep disturbance (11.68%), 540 had gout (5.01%), 462 had COPD (4.28%), 92 had AF (0.85%), 77 had depression (0.71%), 67 had RA (0.62%), 53 had LVH (0.49%), 18 had hyperthyroidism (0.17%), and 2,347 had DN (21.77%).

**Table 1:** Baseline characteristics of solid organ transplant recipients after matching.

Variables	Total		Comparison		SOT recipients		p-value
	N	%	N	%	N	%	
Total	53,915	100.00	43,132	80.00	10,783	20.00	
Sex ^a^							1.000
Female	20,730	38.45	16,584	38.45	4,146	38.45	
Male	33,185	61.55	26,548	61.55	6,637	61.55	
Age (year) ^a^							1.000
≤40	14,055	26.07	11,243	26.07	2,812	26.08	
41-50	13,894	25.77	11,117	25.77	2,777	25.75	
51-60	17,957	33.31	14,364	33.30	3,593	33.32	
≥61	8,009	14.85	6,408	14.86	1,601	14.85	
Mean ± SD	48.47 ± 15.15		48.80 ± 15.30		47.12 ± 14.44		
Insured salary (NTD) ^a^							1.000
≤21,000	24,149	44.79	19,320	44.79	4,829	44.78	
21,001-33,000	13,722	25.45	10,976	25.45	2,746	25.47	
≥33,001	16,044	29.76	12,836	29.76	3,208	29.75	
Urbanization ^a^							1.000
Level 1	15,183	28.16	12,144	28.16	3,039	28.18	
Level 2	17,729	32.88	14,184	32.89	3,545	32.88	
Level 3	9,175	17.02	7,340	17.02	1,835	17.02	
Level 4	7,301	13.54	5,839	13.54	1,462	13.56	
Level 5	864	1.60	693	1.61	171	1.59	
Level 6	1,735	3.22	1,388	3.22	347	3.22	
Level 7	1,928	3.58	1,544	3.58	384	3.56	
CCI score ^a^							1.000
0	2,251	4.18	1,800	4.17	451	4.18	
1	2,214	4.11	1,772	4.11	442	4.10	
2	11,525	21.38	9,220	21.38	2,305	21.38	
≥3	37,925	70.34	30,340	70.34	7,585	70.34	
Enrolled year ^a^							1.000
2002	2,060	3.82	1,648	3.82	412	3.82	
2003	2,295	4.26	1,836	4.26	459	4.26	
2004	3,500	6.49	2,800	6.49	700	6.49	
2005	4,155	7.71	3,324	7.71	831	7.71	
2006	4,870	9.03	3,896	9.03	974	9.03	
2007	4,025	7.47	3,220	7.47	805	7.47	
2008	4,510	8.37	3,608	8.37	902	8.37	
2009	3,825	7.09	3,060	7.09	765	7.09	
2010	3,815	7.08	3,052	7.08	763	7.08	
2011	4,045	7.50	3,236	7.50	809	7.50	
2012	3,850	7.14	3,080	7.14	770	7.14	
2013	4,370	8.11	3,496	8.11	874	8.11	
2014	4,310	7.99	3,448	7.99	862	7.99	
2015	4,285	7.95	3,428	7.95	857	7.95	
Comorbidities							
HTN							<0.001
No	34,607	64.19	28,916	67.04	5,691	52.78	
Yes	19,308	35.81	14,216	32.96	5,092	47.22	
LVH							<0.001
No	53,738	99.67	43,008	99.71	10,730	99.51	
Yes	177	0.33	124	0.29	53	0.49	
AF							0.132
No	53,386	99.02	42,695	98.99	10,691	99.15	
Yes	529	0.98	437	1.01	92	0.85	
HPL							<0.001
No	41,885	77.69	32,769	75.97	9,116	84.54	
Yes	12,030	22.31	10,363	24.03	1,667	15.46	
RA							<0.001
No	53,131	98.55	42,415	98.34	10,716	99.38	
Yes	784	1.45	717	1.66	67	0.62	
Depression							<0.001
No	53,329	98.91	42,623	98.82	10,706	99.29	
Yes	586	1.09	509	1.18	77	0.71	
Sleep disturbance							0.092
No	47,364	87.85	37,840	87.73	9,524	88.32	
Yes	6,551	12.15	5,292	12.27	1,259	11.68	
Gout							0.286
No	51,321	95.19	41,078	95.24	10,243	94.99	
Yes	2,594	4.81	2,054	4.76	540	5.01	
COPD							<0.001
No	49,336	91.51	39,015	90.45	10,321	95.72	
Yes	4,579	8.49	4,117	9.55	462	4.28	
Hyperthyroidism							0.089
No	53,852	99.88	43,087	99.90	10,765	99.83	
Yes	63	0.12	45	0.10	18	0.17	
CKD							<0.001
No	51,420	95.37	42,760	99.14	8,660	80.31	
Yes	2,495	4.63	372	0.86	2,123	19.69	
DM							<0.001
No	38,844	72.05	30,408	70.50	8,436	78.23	
Yes	15,071	27.95	12,724	29.50	2,347	21.77	

a Variables for propensity score matching.

SOT, solid organ transplant recipients; CCI, Charlson comorbidity index; HTN, hypertension; LVH, left ventricular hypertrophy; AF, atrial fibrillation; HPL, hyperlipidemia; RA, rheumatoid arthritis; COPD, chronic obstructive pulmonary disease; CKD, chronic kidney disease; DM, diabetes mellitus.

**Table 2** presents the double-variable analysis for each variable and the incidence of osteoporosis and pathological fracture. Among SOT recipients, 412 (3.82%) developed osteoporosis (incidence rate: 4.13 per 1,000 person-years), and the incidence rate was significantly higher than the comparison cohort (*p* < 0.001). Pathological fractures occurred in 241 (2.23%) of SOT recipients (incidence rate: 2.37 per 1,000 person-years), and the incidence rate was also significantly higher than the comparison cohort (*p* = 0.014). Compared with patients without comorbidities, those with HTN, HPL, RA, sleep disturbance, COPD, and CKD had a higher incidence rate of osteoporosis, and those with HTN, LVH, RA, sleep disturbance, gout, COPD, CKD, and DM had a higher incidence rate of pathological fracture (all *p* < 0.05).

**Table 2:** The incidence rate of osteoporosis and pathological fracture.

Variables	Incident osteoporosis						Incident pathological fracture					
	No		Yes		IR ^a^	p-value	No		Yes		IR ^a^	p-value
	N	%	N	%			N	%	N	%		
Total	52,338	97.08	1,577	2.92	3.14		52,868	98.06	1,047	1.94	2.06	
Patients						<0.001						0.014
Comparison	41,967	97.30	1,165	2.70	2.89		42,326	98.13	806	1.87	1.98	
SOT recipients	10,371	96.18	412	3.82	4.13		10,542	97.77	241	2.23	2.37	
Kidney transplant	6,135	95.89	263	4.11	4.09		6,268	97.97	130	2.03	1.98	
Liver transplant	4,166	96.64	145	3.36	4.17		4,206	97.56	105	2.44	2.98	
Heart/Lung transplant	70	94.59	4	5.41	6.00		68	91.89	6	8.11	9.36	
Sex						<0.001						<0.001
Female	19,684	94.95	1,046	5.05	5.28		20,152	97.21	578	2.79	2.86	
Male	32,654	98.40	531	1.60	1.74		32,716	98.59	469	1.41	1.53	
Age (year)						<0.001						<0.001
≤40	13,965	99.36	90	0.64	0.63		13,986	99.51	69	0.49	0.48	
41-50	13,588	97.80	306	2.20	2.24		13,712	98.69	182	1.31	1.32	
51-60	17,255	96.09	702	3.91	4.45		17,551	97.74	406	2.26	2.53	
≥61	7,530	94.02	479	5.98	7.31		7,619	95.13	390	4.87	5.84	
Mean ± SD	48.20 ± 15.14		57.37 ± 12.38				48.26 ± 15.10		59.02 ± 13.54			
Insured salary						<0.001						0.009
≤21,000	23,316	96.55	833	3.45	3.33		23,634	97.87	515	2.13	2.03	
21,001-33,000	13,405	97.69	317	2.31	2.98		13,465	98.13	257	1.87	2.40	
≥33,001	15,617	97.34	427	2.66	2.93		15,769	98.29	275	1.71	1.86	
Urbanization						<0.001						0.011
Level 1	14,774	97.31	409	2.69	2.74		14,903	98.16	280	1.84	1.86	
Level 2	17,127	96.60	602	3.40	3.65		17,393	98.10	336	1.90	2.01	
Level 3	8,951	97.56	224	2.44	2.71		9,015	98.26	160	1.74	1.92	
Level 4	7,094	97.16	207	2.84	3.09		7,150	97.93	151	2.07	2.23	
Level 5	842	97.45	22	2.55	2.97		842	97.45	22	2.55	2.96	
Level 6	1,669	96.20	66	3.80	4.31		1,693	97.58	42	2.42	2.71	
Level 7	1,881	97.56	47	2.44	2.82		1,872	97.10	56	2.90	3.34	
CCI score						<0.001						<0.001
0	2,210	98.18	41	1.82	1.74		2,227	98.93	24	1.07	1.01	
1	2,166	97.83	48	2.17	2.03		2,187	98.78	27	1.22	1.13	
2	11,208	97.25	317	2.75	2.73		11,354	98.52	171	1.48	1.45	
≥3	36,754	96.91	1,171	3.09	3.45		37,100	97.82	825	2.18	2.40	
Comorbidities												
HTN						<0.001						<0.001
No	33,746	97.51	861	2.49	2.58		34,053	98.40	554	1.60	1.64	
Yes	18,592	96.29	716	3.71	4.24		18,815	97.45	493	2.55	2.88	
LVH						0.937						0.013
No	52,166	97.07	1,572	2.93	3.14		52,699	98.07	1,039	1.93	2.05	
Yes	172	97.18	5	2.82	3.14		169	95.48	8	4.52	5.04	
AF						0.152						0.070
No	51,830	97.09	1,556	2.91	3.12		52,355	98.07	1,031	1.93	2.05	
Yes	508	96.03	21	3.97	4.90		513	96.98	16	3.02	3.69	
HPL						0.010						0.580
No	40,702	97.18	1,183	2.82	2.95		41,079	98.08	806	1.92	1.99	
Yes	11,636	96.72	394	3.28	3.88		11,789	98.00	241	2.00	2.34	
RA						<0.001						<0.001
No	51,605	97.13	1,526	2.87	3.08		52,112	98.08	1,019	1.92	2.03	
Yes	733	93.49	51	6.51	6.91		756	96.43	28	3.57	3.70	
Depression						0.481						0.678
No	51,772	97.08	1,557	2.92	3.13		52,292	98.06	1,037	1.94	2.06	
Yes	566	96.59	20	3.41	4.10		576	98.29	10	1.71	2.03	
Sleep disturbance						<0.001						<0.001
No	46,080	97.29	1,284	2.71	2.88		46,514	98.21	850	1.79	1.89	
Yes	6,258	95.53	293	4.47	5.13		6,354	96.99	197	3.01	3.40	
Gout						0.194						0.004
No	49,809	97.05	1,512	2.95	3.16		50,344	98.10	977	1.90	2.02	
Yes	2,529	97.49	65	2.51	2.71		2,524	97.30	70	2.70	2.91	
COPD						<0.001						<0.001
No	47,966	97.22	1,370	2.78	2.97		48,423	98.15	913	1.85	1.96	
Yes	4,372	95.48	207	4.52	4.90		4,445	97.07	134	2.93	3.12	
Hyperthyroidism						0.387						0.264
No	52,278	97.08	1,574	2.92	3.13		52,805	98.06	1,047	1.94	2.06	
Yes	60	95.24	3	4.76	5.59		63	100.00	–	–	–	
CKD						0.002						0.031
No	49,943	97.13	1,477	2.87	3.10		50,436	98.09	984	1.91	2.04	
Yes	2,395	95.99	100	4.01	3.85		2,432	97.47	63	2.53	2.38	
DM						0.357						0.014
No	37,724	97.12	1120	2.88	2.99		38,125	98.15	719	1.85	1.90	
Yes	14,614	96.97	457	3.03	3.55		14,743	97.82	328	2.18	2.52	

a The incidence rate of per 1,000 person-years.

SOT, solid organ transplant recipients; CCI, Charlson comorbidity index; HTN, hypertension; LVH, left ventricular hypertrophy; AF, atrial fibrillation; HPL, hyperlipidemia; RA, rheumatoid arthritis; COPD, chronic obstructive pulmonary disease; CKD, chronic kidney disease; DM, diabetes mellitus.

After other relevant influencing factors were controlled for, SOT recipients had a higher risk of osteoporosis (HR: 1.46, 95% CI: 1.29–1.65) than controls (**Table 3**). Compared with patients <40 years old, the risk of osteoporosis was higher in those aged 41 to 50 years (HR: 3.49, 95% CI: 2.76–4.42), 51 to 60 years (HR: 7.08, 95% CI: 5.66–8.86), and ≥60 years (HR: 11.51, 95% CI: 9.10–14.56). Patients with a CCI score of >3 had a higher risk of osteoporosis than those with a CCI score of 0. Compared with patients without comorbidities, patients with RA (HR: 1.57, 95% CI: 1.18–2.07), sleep disturbance (HR: 1.27, 95% CI: 1.12–1.45), and COPD (HR: 1.35, 95% CI: 1.16–1.57) had a higher risk of osteoporosis. In comparison, patients with DM had a lower risk of osteoporosis (HR: 0.87, 95% CI: 0.77–0.98). We observed that kidney and liver transplant recipients had a higher risk of osteoporosis (HR: 1.45, 95% CI: 1.24–1.70 and HR: 1.46, 95% CI: 1.22–1.74, respectively).

**Table 3:** Risk of incident osteoporosis and pathological fracture.

Variables	Osteoporosis					Pathological fracture				
	aHR ^1^	95% CI			p-value	aHR ^1^	95% CI			p-value
Patients										
Comparison (ref.)	1					1				
Organ transplant	1.46	1.29	–	1.65	<0.001	1.19	1.01	–	1.39	0.034
Kidney transplant	1.45	1.24	–	1.70	<0.001	0.94	0.75	–	1.17	0.561
Liver transplant	1.46	1.22	–	1.74	<0.001	1.44	1.17	–	1.78	<0.001
Heart/Lung transplant	1.73	0.64	–	4.64	0.279	4.62	2.05	–	10.44	<0.001
Gender										
Female (ref.)	1					1				
Male	0.30	0.27	–	0.34	<0.001	0.47	0.41	–	0.53	<0.001
Age (year)										
≤40 (ref.)	1					1				
41-50	3.49	2.76	–	4.42	<0.001	2.64	2.00	–	3.50	<0.001
51-60	7.08	5.66	–	8.86	<0.001	5.25	4.04	–	6.82	<0.001
≥61	11.51	9.10	–	14.56	<0.001	11.99	9.16	–	15.69	<0.001
Insured salary										
≤21,000 (ref.)	1					1				
21,001-33,000	0.84	0.73	–	0.95	0.007	1.25	1.07	–	1.45	0.005
≥33,001	0.96	0.85	–	1.08	0.453	1.02	0.87	–	1.18	0.843
Urbanization										
Level 1 (ref.)	1					1				
Level 2	1.35	1.19	–	1.53	<0.001	1.10	0.94	–	1.29	0.255
Level 3	1.16	0.98	–	1.36	0.084	1.21	0.99	–	1.47	0.058
Level 4	1.22	1.03	–	1.44	0.021	1.26	1.03	–	1.54	0.022
Level 5	1.10	0.72	–	1.69	0.665	1.61	1.04	–	2.49	0.032
Level 6	1.50	1.16	–	1.95	0.002	1.39	1.00	–	1.93	0.048
Level 7	1.06	0.78	–	1.43	0.730	1.76	1.32	–	2.35	<0.001
CCI score ^2^										
0 (ref.)	1					1				
1	1.21	0.80	–	1.84	0.366	1.11	0.64	–	1.93	0.705
2	1.42	1.02	–	1.97	0.036	1.36	0.89	–	2.10	0.159
≥3	1.54	1.13	–	2.12	0.007	1.80	1.19	–	2.72	0.005
Comorbidities (Yes vs no)										
HTN ^2^	1.06	0.95	–	1.18	0.310	1.06	0.93	–	1.21	0.376
LVH ^2^	0.74	0.31	–	1.78	0.497	1.72	0.86	–	3.46	0.126
AF ^2^	0.97	0.63	–	1.49	0.875	0.99	0.60	–	1.64	0.982
HPL ^2^	1.05	0.93	–	1.18	0.478	0.90	0.77	–	1.05	0.178
RA ^2^	1.57	1.18	–	2.07	0.002	1.34	0.92	–	1.95	0.133
Depression	1.03	0.66	–	1.61	0.892	0.76	0.41	–	1.43	0.400
Sleep disturbance	1.27	1.12	–	1.45	<0.001	1.36	1.16	–	1.59	<0.001
Gout	0.95	0.74	–	1.22	0.668	1.40	1.09	–	1.79	0.008
COPD ^2^	1.35	1.16	–	1.57	<0.001	1.13	0.94	–	1.36	0.195
Hyperthyroidism	1.42	0.45	–	4.42	0.549	–		–		–
CKD ^2^	1.00	0.80	–	1.24	0.963	1.04	0.79	–	1.38	0.782
DM ^2^	0.87	0.77	–	0.98	0.025	0.92	0.80	–	1.06	0.265

^1^ aHR, adjusted hazard ratio, that estimates in Cox proportional hazards model.

^2^ SOT, solid organ transplant recipients; CCI, Charlson comorbidity index; HTN, hypertension; LVH, left ventricular hypertrophy; AF, atrial fibrillation; HPL, hyperlipidemia; RA, rheumatoid arthritis; COPD, chronic obstructive pulmonary disease; CKD, chronic kidney disease; DM, diabetes mellitus.

SOT recipients had a higher risk of pathological fracture HR of 1.19 (95% CI: 1.01–1.39) than controls. Compared with patients <40 years old, the risk of pathological fracture was higher in those aged 41 to 50 years (HR: 2.64, 95% CI: 2.00–3.50), 51 to 60 years (HR: 5.25, 95% CI: 4.04–6.82), and ≥60 years (HR: 11.99, 95% CI: 9.16–15.69). Patients with a CCI score of >3 had a higher risk of pathological fracture than those with a CCI score of 0. Patients with sleep disturbance had a higher risk of pathological fracture (HR: 1.36, 95% CI: 1.16–1.59). Patients with gout had a higher risk of pathological fracture (HR: 1.40, 95% CI: 1.09–1.79). We observed that liver and heart or lung transplant recipients had a higher risk of pathological fracture (HR: 1.44, 95% CI: 1.17–1.78 and HR: 4.62, 95% CI: 2.05–10.44, respectively).

## Discussion

Our main findings were that SOT recipients had a higher risk of osteoporosis and associated pathological fractures than the general population. To compare the risk of different recipients of SOT, the highest risk of fractures was noted in patients receiving heart or lung transplants. We also found that SOT recipients comorbid with RA, sleep disturbances, gout, and COPD had a higher risk of osteoporosis or fractures. In addition, SOT recipients who scored high on the CCI had a high HR for developing osteoporosis and fractures, and women had a higher risk than men.

Osteoporosis is a frequent and devastating complication after SOT (18) and is caused by multiple pathophysiologic mechanisms (7). Increased risk of posttransplant fracture in SOT recipients is mainly due to posttransplant bone remodeling due to reduced bone formation and continued bone erosion resulting in bone mineralization (19). Posttransplantation osteoporosis and fracture are associated with alterations in the receptor activator of the nuclear factor B ligand (RANKL)/osteoprotegerin (OPG) system (18) (20),. OPG is an antiresorptive cytokine that competes with RANKL for binding to RANK and inhibits osteoclast differentiation. OPG plays a central role in the development of transplantation osteoporosis and fracture (21).

In our study, SOT recipients had a higher risk of osteoporosis and related fractures than the general population. The loss of bone mass is particularly prominent during the first 3 to 6 months after heart, lung, and liver transplantation and during the first 6 to 18 months after kidney transplantation (22). The reduction in BMD varies between 2% and 12% in the first posttransplant year in all transplantations (23). A wide range of osteoporosis (11%–57%) and fracture rates (14%–65%) have been reported during the first posttransplant year, depending on the transplant organ and follow-up duration (24–26).

Some previous studies have shown that kidney transplant recipients have a higher risk of fracture than the general population (11, 27, 28). However, in our cohort study, kidney transplant patients had a higher risk of osteoporosis than the general population but not fracture risk. A Canadian cohort study using healthcare databases also demonstrated that the cumulative incidence of fracture in kidney transplant recipients was lower than in previously reported literature (29). This study suggested that despite the metabolic bone changes and use of steroids following kidney transplantation, recipients may not be a high-risk group for fracture. Explanations for the lower-than-expected fracture incidence may be associated with changes in maintenance immunosuppressive regimens (29). There has also been a trend toward decreasing corticosteroid doses after kidney transplantation. Corticosteroids are well known to be associated with a decrease in bone mineral density (30).

Posttransplantation bone disease is a major cause of morbidity among kidney transplant recipients, with a significantly higher risk of subsequent fractures, which are associated with a greater increase in mortality rates (31). Within the first 5 years after transplantation, an estimated 22.5% of kidney transplant recipients experience a fracture, an incidence that was four times higher in the general population (32). This risk remains significantly elevated in transplant recipients even after 10 years, suggesting that biochemical abnormalities of disordered mineral metabolism are common in recipients with kidney failure and may persist after a successful renal transplant (31).

We can also find that liver transplant recipients had a higher risk of osteoporosis and pathological fractures than the general population. Studies have concluded that most liver transplant recipients already have abnormal BMD at the time of transplantation or have had fractures before the transplant (33, 34). BMD may improve mainly in the second year after surgery, but approximately one-third of liver transplant recipients’ BMD remains under the fracture threshold (35). Low BMD before transplantation is a major risk factor for posttransplant fractures (36). The reason for BMD reduction in the early posttransplant period may be due to the pre- and posttransplant changes in bone turnover state. A meta-analysis indicated that liver transplant recipients have a fivefold increased risk of both osteoporosis and fractures compared with non-LT recipients (37). The main risk factors for postliver transplant fractures include the presence of pretransplant fractures, decreased BMD, posttransplant glucocorticoid dose, and primary biliary cholangitis (38).

Among the recipients of different SOTs, the highest risk of fractures was noted in patients receiving heart or lung transplants. Bone mass loss and fragility fractures are important complications after a heart transplant. Significant bone loss occurs after heart transplantation, and is associated with an increased risk of fracture-related morbidity and mortality rates (1). In heart transplant recipients, the incidence of fractures was highest in the first year after transplantation (39). Lung transplant recipients have a higher risk of osteoporosis and pathologic fractures than kidney, liver, and heart transplant recipients (13). This may be because lung transplant recipients are required more intensive administration of high doses of immunosuppressants to improve patient survival (40).

Our study revealed that patients with higher scores on the CCI were also at higher risk, especially those with CCI scores of >3. In addition, women had a higher risk than men. Many factors contribute to the development of bone disease and osteoporosis after SOT, including immunosuppressive agents and lifestyle risk factors (7). Typically, older adults, women, and recipients of dialysis have an increased risk of fracture (28). CCI is associated with increased mortality in fragility fracture patients (41). The CCI score may be applicable for predicting fracture risk.

We found that patients comorbid with RA, sleep disturbances, gout, and COPD had a higher risk of developing osteoporosis or fracture. The prevalence of osteoporosis in RA is approximately 30% (up to 50% in postmenopausal women), which might be a twofold increase over the general population (42, 43).

A cross-sectional study indicated that bone density is associated with sleep quality and duration (44). Sleep disorders are associated with an increased risk of osteoporosis, especially in women and older adults (45). A population-based epidemiologic study indicated that gout modestly increases the risk of osteoporosis (46). Bone disorders can develop before any organ transplantation; however, they seem to be pronounced in patients with chronic lung diseases (47, 48). COPD is associated with an increased risk of osteoporosis, regardless of the exposure period (49). Osteoporosis is highly prevalent in patients referred for lung transplantation, especially among patients with COPD (4). Evidence regarding vertebral fracture (VF) risk in patients with T2DM has been less conclusive, providing results ranging from lower risk to no association with higher prevalent or incident VF to increased risk (50–55). However, our study discovered that patients comorbid with T2DM had a lower risk of developing osteoporosis but no association with fracture. In a sample of 37,292 patients with T2DM, there was a lower risk of prevalent VF (OR: 0.84, 95% CI: 0.74–0.95) without evidence of heterogeneity across studies. In a sample of 738,018 patients with T2DM, there was an increased risk of incident VF (OR: 1.35, 95% CI: 1.27–1.44) with no evidence of heterogeneity across studies (56).

The present study has several strengths. First, we included the entire Taiwanese population in this study; thus, the sample size is large and highly representative of the general population, thus increasing statistical power. The combination of NHIRD with multiple data sources can be a powerful research tool. The large-scale nationwide data include comprehensive demographic data and long-term follow-up duration. Second, we also investigated related risk factors for comorbidities and the risk of incident osteoporosis and fracture following transplantation.

Our study also had certain limitations. First, dual-energy X-ray absorptiometry (DXA) is currently considered the gold standard for the assessment of BMD and, thus, osteoporosis diagnosis. However, the NHIRD does not contain data on bone density Z-scores or T-scores, precluding the confirmation of osteoporosis diagnosis. Second, the NHIRD does not contain detailed information regarding family history of systemic diseases and socioeconomic status, which may be risk factors for osteoporosis or pathologic fracture. Finally, the NHIRD also lacks information on other risk factors for osteoporosis and osteoporotic fractures, such as body mass index, smoking, vitamin D intake, calcium intake, diet supplementation, and physical activity.

## Conclusion

SOT recipients had higher incidence rates and risks of osteoporosis and fractures than the general population. Among different recipients of SOT, the highest risk of fractures was noted in patients receiving heart or lung transplants. In addition, patients who scored high on the CCI had a higher risk. Patients who were comorbid with RA, sleep disturbance, gout, and COPD had a higher risk of osteoporosis or fractures.

## Data availability statement

The National Health Insurance Database used to support the findings of this study was provided by the Health and Welfare Data Science Center, Ministry of Health and Welfare (HWDC, MOHW) under license and cannot be made freely available. Requests for access to these data should be made to HWDC (https://dep.mohw.gov.tw/dos/np-2497-113.html).

## Ethics statement

In the present study, we conducted secondary data analysisusing the Longitudinal Health Insurance Database (LHID) released by the Health and Welfare Data Science Center (HWDC) at the Ministry of Health and Welfare in Taiwan. The LHID is anonymous, and the HWDC provides scrambled random identification numbers for insured patients to protect their privacy. The requirement for informed consent was waived. This study protocol was approved as an ethical review by the Central Regional Research Ethics Committee of China Medical University, Taiwan (No. CRREC-109-011).

## Author contributions

Study conception and design: K-HH, HC, Y-RL, YY, S-YG, C-YH, T-HT, and C-YL. Data acquisition: K-HH and C-YL. Data analysis and demonstration: K-HH and C-YL. Original draft preparation: K-HH, Y-RL, YY, HC, S-YG, T-HT, C-YH, and C-YL. All authors contributed to the article and approved the submitted version.
